# Finger-actuated microfluidic device for point-of-care circulating cell-free nucleic acid extraction

**DOI:** 10.1039/d6lc00439c

**Published:** 2026-08-14

**Authors:** Ghulam Musaddaq, Labibah Ehsan, Yano Yoga, Joshua Castillo, Linda Marriott, Nicholas R. Leslie, Deborah Williamson, Thomas Tran, Chuan Kok Lim, Maïwenn Kersaudy-Kerhoas

**Affiliations:** a School of Engineering and Physical Sciences, Heriot-Watt University UK m.kersaudy-kerhoas@hw.ac.uk; b Middle East Technical University Türkiye; c Victorian Infectious Diseases Reference Laboratory at the Peter Doherty Institute for Infection and Immunity, Royal Melbourne Hospital Australia; d The University of St Andrews UK

## Abstract

Timely and accurate nucleic acid extraction is a critical bottleneck in point-of-care (POC) molecular diagnostics, particularly for low-abundance cell-free nucleic acids (DNA, RNA, miRNA) in plasma. We present a fully finger-actuated microfluidic device for power-free, pipette-free, nucleic acid extraction. The prototype device integrates finger-actuated valves, pump and blister reagent storage, enabling a complete, intuitive, “sample-to-eluate” workflow, including lysis, binding, washing and elution *via* manual actuation in approximately 35 minutes. The device operation was benchmarked against a commercial magnetic bead-based manual benchtop and an automated robotic workflow. In DNA-spiked aqueous samples (5–20 ng mL^−1^), the device demonstrated recoveries of 84.6%, against 90.7% on the bench. Optimisation of magnetic bead volume identified 50 μL as the threshold for maximal recovery while minimising reagent consumption. Recovery from human pooled plasma samples were found to be on average 18, 62, and 552% higher than that obtained with the control bench extraction on initial sample volumes of 100, 75 and 50 μL, respectively. Coefficient of variation (CV%) for all techniques remained within reported laboratory ranges (20–80%). The device's clinical utility was piloted using patient samples positive for cytomegalovirus (CMV), monkeypox virus (MPXV) or chikungunya (CHIKV). The device achieved successful qPCR amplification for all targets, with viral DNA Cq values within as little as 0.3 cycles of the standard protocol. This instrument-free, manual circulating nucleic acid extraction tool could help bridge the gap between lateral flow based nucleic acid amplification technologies and real-world decentralised diagnostics, offering a scalable solution for infectious disease management in resource-limited settings.

## Abstract 2

Timely and accurate nucleic acid extraction is a critical bottleneck in point-of-care (POC) molecular diagnostics, particularly for low-abundance circulating cell-free nucleic acids (DNA, RNA, miRNA) in plasma.

## Graphical abstract

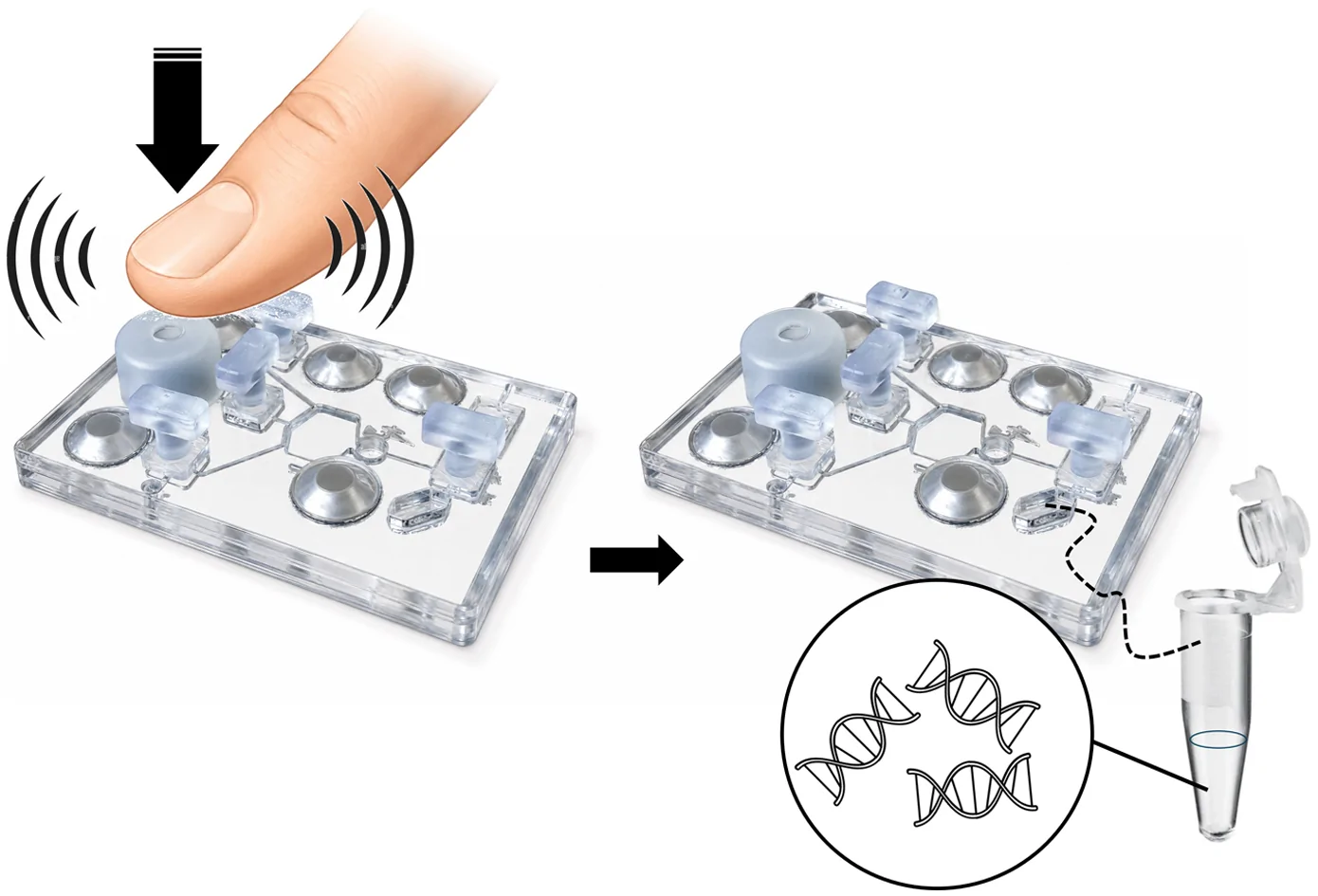

## Introduction

Accurate and timely diagnosis is fundamental to effective healthcare delivery. Molecular diagnostics, particularly nucleic acid amplification tests (NAATs), have become indispensable tools for the detection of infectious diseases, metabolic disorders and cancers due to their high analytical sensitivity and specificity.^1,2^ Widely adopted NAAT platforms include polymerase chain reaction (PCR), loop-mediated isothermal amplification (LAMP), and more recently, CRISPR-Cas-based detection systems, each offering distinct trade-offs between speed, sensitivity and operational complexity.^3^ Many of these platforms are now coupled with visual readouts, such as lateral flow assays (LFAs), enhancing their suitability for point-of-care (POC) and field-based applications.^4^ The adaptability of these systems was exemplified during the COVID-19 pandemic, when CRISPR-based assays were rapidly authorised for emergency use by the FDA.^5^

Despite these advances, LFA-NAAT performance remains critically dependent on the availability of high-quality, purified nucleic acids, positioning upstream sample preparation as a major limitation in decentralised diagnostics.^6^ Circulating cell-free nucleic acids (cfNAs), including cell-free DNA (cfDNA) and microRNAs (miRNAs), are increasingly recognised as minimally invasive biomarkers for conditions such as liver injury, cancer and sepsis.^7^ Their relative stability in plasma and disease-specific signature expression profiles make them attractive targets for liquid biopsy-based diagnostics; however, rapid and efficient extraction of cfNAs from blood plasma remains technically challenging, particularly outside conventional laboratory environments.^8^

Current nucleic acid extraction workflows rely predominantly on silica-membrane or magnetic bead-based chemistries and necessitate multiple sequential steps. These protocols are reagent-intensive, requiring precise pipetting and periodic centrifugation or vacuum steps; consequently, they remain tethered to trained field personnel or centralised laboratory infrastructure.^9–11^ This infrastructure dependency represents a critical bottleneck in infectious disease management, where complex testing is therefore largely restricted to formal healthcare settings. As a result, sample preparation often dictates the total diagnostic turnaround time while introducing significant variability, contamination risks and inconsistent yields.^12^ Such constraints preclude diagnostic access for remote populations, vulnerable groups and patients in home isolation, all of whom require ‘true’ point-of-care (POC) solutions capable of self-testing.^13^ These logistical barriers significantly limit the translation of advanced LFA-NAATs to POC settings, particularly in resource-limited or field settings.

To address these challenges, there is growing interest in integrated sample-to-answer platforms that combine nucleic acid extraction, amplification and detection within a single, user-friendly format. Point-of-care diagnostics have already transformed healthcare delivery through technologies such as home pregnancy tests, glucose meters and very familiar COVID-19 antigen tests.^14,15^ In this context, microfluidic systems offer a compelling route towards miniaturised and scalable nucleic acid extraction at the POC. However, fully integrated extraction platforms are largely confined to laboratory-scale demonstrations such as BioFire Filmarray, and others, whilst solutions that are low-cost, power-free and operable by non-experts remain limited.^16,17^

Self-operated microfluidic platforms aim to eliminate external instrumentation by exploiting passive or manually driven flow mechanisms. Among these, ‘finger-actuated’ microfluidic solutions have emerged as a promising approach for decentralised diagnostics, enabling fluid manipulation through direct finger actuation without the need for electrical pumps or pressure controllers while maintaining simplicity and robustness.^18,19^ Finger-actuated mechanisms range from manual turning valves to mechanical air cap pumps,^20^ pull-cord-driven centrifugal discs^21^ and finger-pierceable blister reagent storage modules.^22^ These approaches significantly reduce system complexity and manufacturing costs, making them well-suited for low-resource, field-deployable applications. Such finger-actuated microfluidic systems have been successfully developed to enable the rapid, POC detection of respiratory viruses, including SARS-CoV-2 and influenza A.^23^ Recently, Park *et al.* demonstrated a finger-actuated device for nucleic acid purification. The PDMS device featured an integrated finger-pump which resulted in a 50% nucleic acid recovery rate and demonstrated positive HBV identification on four patient plasma samples.^24^

We have previously demonstrated a finger-actuated microfluidic device capable of generating filtered blood lysate in under three minutes using modular components including 3D printed finger-actuated valves, a mechanical air-cap pump and blister-based reagent storage.^25^ The device's intuitive operation was anecdotally demonstrated by one of the authors' daughter demonstrating its use with just eight lines of instructions.^26^ Building on this work, we present a standalone finger-actuated microfluidic platform for nucleic acid extraction from plasma samples. The core concept of this platform is to substitute conventional, instrument-dependent benchtop cfNA extraction protocols with an intuitive, simple, finger-actuated device in a highly portable format.

The proposed device (Fig. 1) uses the same modular approach with custom finger-actuated valves, pump and blister storage to enable controlled, but power-free, cfNA purification without external instrumentation. The extraction performance is demonstrated against the magnetic bead-based bench extraction method, and robotic extraction, demonstrating comparable nucleic acid yields at standard sample input volumes, with scope for further optimisation at reduced sample volumes.

**Fig. 1:**
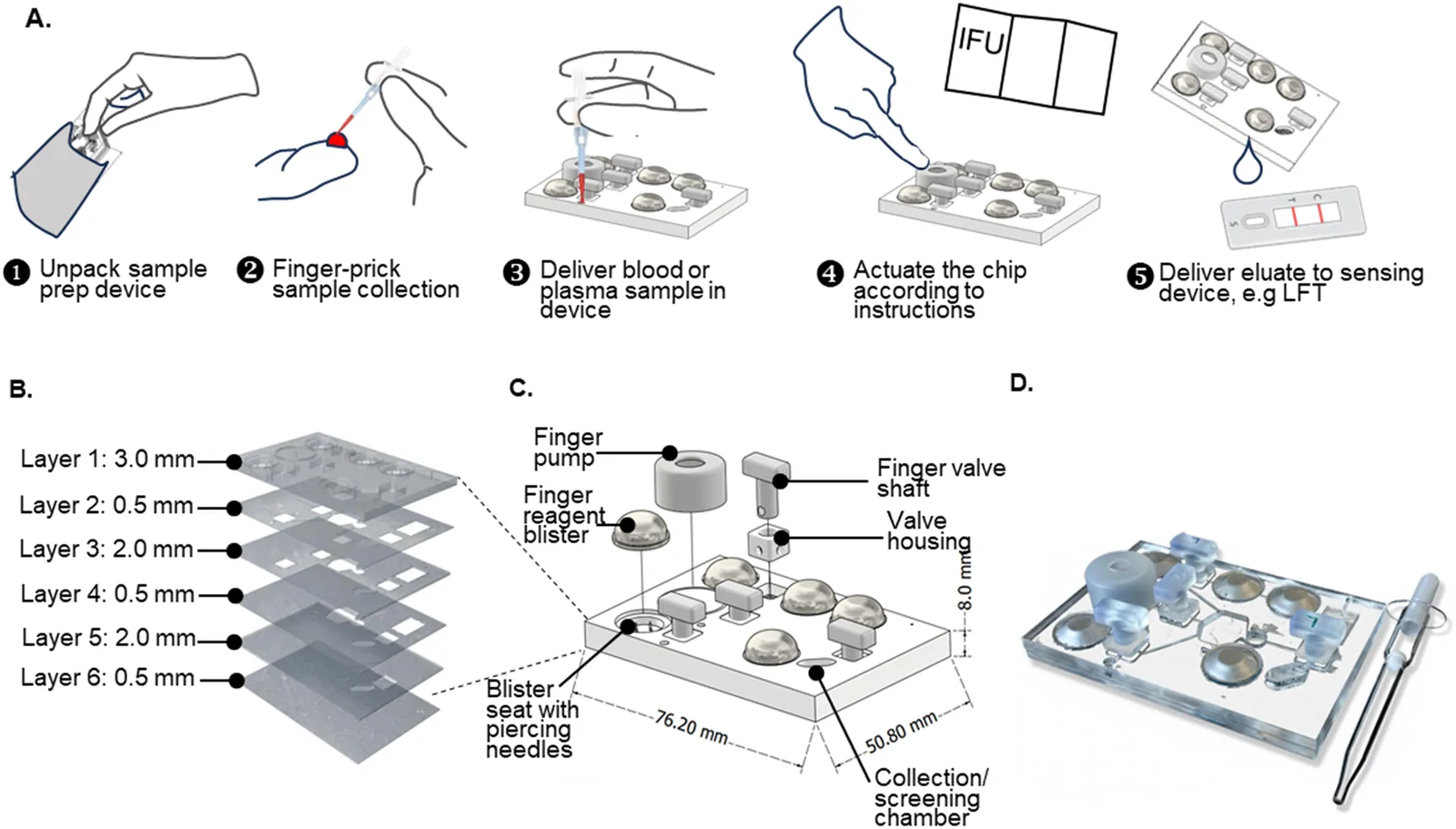
Finger-actuated microfluidic device for cell-free nucleic acid extraction. A. Step-by-step concept workflow. B. Exploded view of the device illustrating the multilayer fabrication strategy. C. Rendered diagram of the device, highlighting the main features including finger-actuated valves, valve housing, reagent-filled blisters and finger-actuated ‘aircap’ pump. D. Photograph of the fully assembled device together with a Sarstedt Minivette® blood collection device.

By addressing the upstream sample preparation bottleneck, this work aims to bridge the gap between advanced NAAT technologies, including CRISPR-based assays and their practical deployment in real-world point-of-care diagnostics.

## Materials and methods

### Device fabrication

The finger-actuated nucleic acid extraction device (Fig. 1C) is composed of six laser-cut layers of polymethyl methacrylate (PMMA, Clarex). Each layer was cut using a CO_2_ laser (Epilog Mini 18, 30 W) to define the fluidic channels, valve interfaces and dedicated chambers for reagent mixing and collection. The six layers were aligned and bonded together using a solvent-assisted thermal bonding technique adapted from Liga *et al.*^27^ In this process, each layer was uniformly sprayed with ethanol, aligned and placed in a heated hydraulic press (Carver). Bonding was performed under a load of 3.5 tonnes at 65 °C for 3 min, resulting in a permanently sealed monolithic device.

Valve shafts, valve housings and the air-cap finger pump used for manual fluid actuation were fabricated by stereolithography (SLA) 3D printing using a Form 2 3D printer (Formlabs). Rigid components *i.e.* valve shafts, were printed using Formlabs Clear Resin (V4), while flexible valve housings and the air-cap pump were fabricated using Elastic 50A Resin to enable compatible actuation. Printed components were post-processed according to the manufacturer's (Formlabs) recommendations and assembled into the bonded PMMA device. A photograph of the fully assembled device along with the Minivette® blood collection device is shown in Fig. 1D. The estimated cost of materials for a single prototype device includes £12.73 of reagents, £9 of custom blister pack filling and £1.81 of other materials. The total of £23.54 represents the material cost of prototypes at very low volume prototype production. Even with added production costs, the cost of a mass-produced device is expected to decrease substantially under high-volume manufacturing conditions. In comparison, a Cepheid cartridge which includes sample preparation and PCR reagents currently retails between £7–20 depending on exact test and country, excluding automation platform costs and annual maintenance. Although the prototyping cost of the finger-actuated device is currently high, it should be appreciated that it has not yet been optimised to reduce costs. Cost-effective, as well as environmentally friendly production of blister pack should be investigated. The total weight of plastics in the device was estimated to be 38.7 g, compared to 6.13 g for the bench method. This included plastics of the reagent bottle (pro-rata), but excluded any equipment materials. Whilst highly convenient for field-deployable and fast cfNA extraction, it is acknowledged that such single-use devices have an environmental impact, and the development of more sustainable solutions should be investigated.

### Device design and operation

The conceptual integrated microfluidic chip (Fig. 2A) incorporates finger-actuated valves and a 3D printed silicone air-cap pump to enable controlled fluid handling without external instrumentation. The sample inlet was designed to interface directly with Minivette® blood collection devices (Sarstedt), eliminating unnecessary pipetting and providing sufficient actuation force to propel a finger-prick blood sample into the device. On-chip reagent storage is achieved using commercial blisters. The blister is pierced by sharp needles engraved directly on the chip, upon simple hand actuation, releasing the required buffer/reagents into the central chamber, as demonstrated in an earlier publication.^25^

**Fig. 2:**
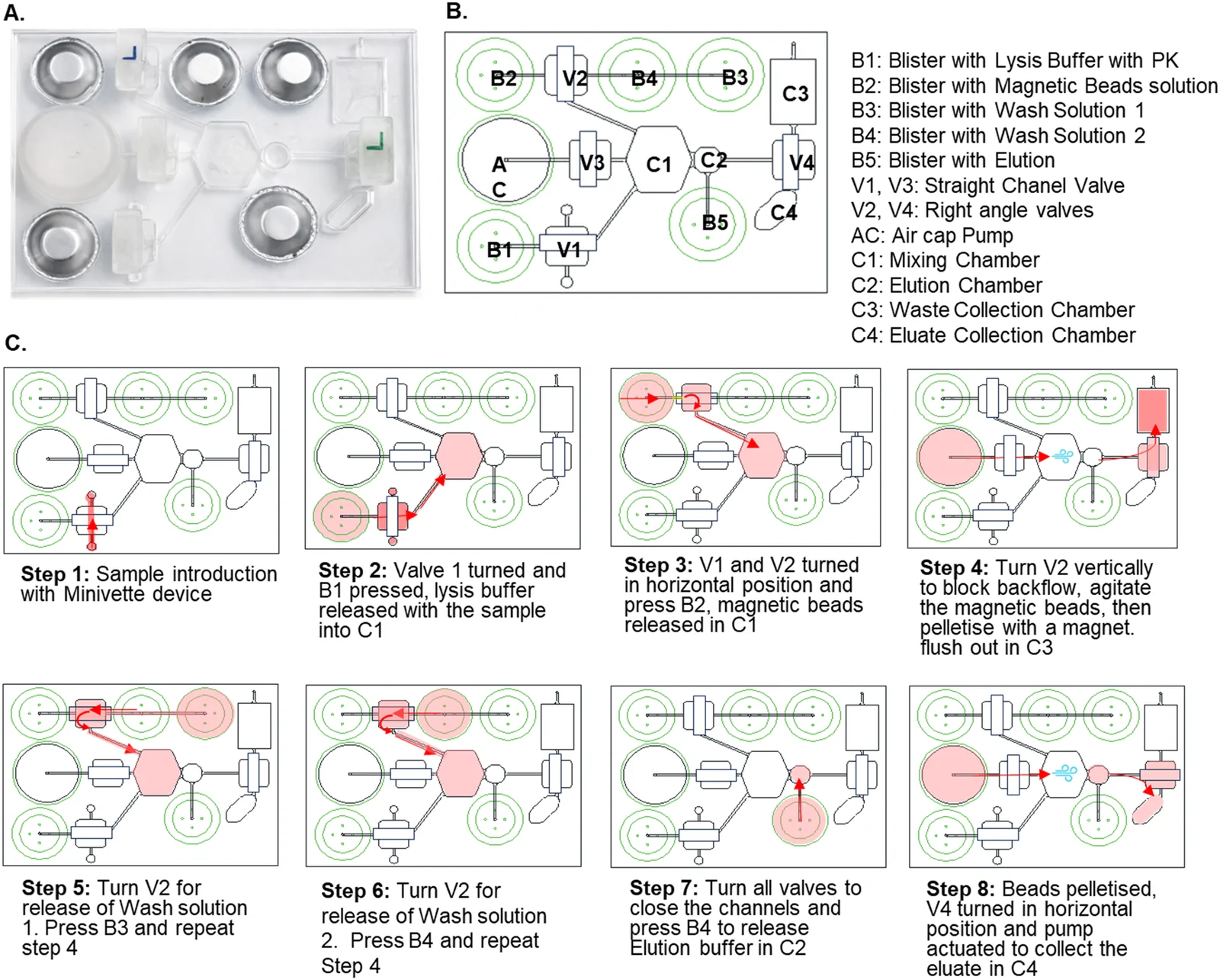
Device operation. A. Top-view photograph of the microfluidic device. B. Schematic layout of the device and key functional units. C. Stepwise operation workflow of the device from sample loading to eluate collection.

### Human samples

Healthy human plasma samples for the device characterisation were sourced from the Scottish National Blood Transfusion Service (contract 18–20; Office for Research Ethics Committees Northern Ireland (ORECNI) ref. 18/NI/0148). To minimise the impact of inter-individual biological variation and ensure sufficient volume for cross-platform benchmarking, plasma samples were pooled into three distinct biological replicates (B011, B012 and B013). This allowed for a direct technical comparison between the finger-actuated device, manual benchtop extraction and automated robotic protocols using identical starting volumes.

Patient samples (plasma samples and lesion swabs) used in the clinical pilot study were obtained from the Victorian Infectious Diseases Reference Laboratory (VIDRL) at the Peter Doherty Institute and collected under ethical approval of Royal Melbourne Hospital Human Research Ethics Committee (RMH HREC 2020.179).

### Bench extraction workflow

To benchmark the finger-actuated device, plasma samples were processed using the MagMAX™ Cell-Free Total Nucleic Acid Isolation Kit (Thermo Fisher Scientific) following the manufacturer's protocol optimised for cfNA extraction from low-volume samples. Briefly, plasma (volumes ranging from 50–100 μL) was first digested with Proteinase K (20 mg mL^−1^, 10 μL) and lysis/binding solution (62.5 μL) at 65 °C for 30 min with agitation (1000 rpm), then cooled to room temperature. Additional lysis/binding solution (93.8 μL) and 50 μL of resuspended magnetic beads were then added to the lysed sample and the suspension was mixed for 10 min to allow nucleic acid binding. Bead–nucleic acid complexes were captured using a magnetic stand, washed sequentially with Wash Solution 1 (200 μL) and Wash Solution 2 (80% ethanol) (200 μL) and air-dried for 5 min. Finally, nucleic acids were eluted in 50 μL elution buffer by vortexing at high speed for 5 min, followed by magnetic separation. The eluate was collected and either used immediately for qPCR or stored at −20 °C. The time required for the bench extraction process is a little over 50 minutes.

### Extraction on finger-actuated device

Plasma input volumes of 50 μL, 75 μL and 100 μL were processed using the finger-actuated microfluidic extraction device. The same reagent formulations and extraction steps described in the bench extraction were adapted for on-chip operation. The device operation workflow (Fig. 2B) consists of 8 sequential steps, from sample loading to eluate recovery, all performed manually by pressing blisters, turning valves and actuating the pump. The whole process took approximately 35 minutes. While the device was designed to support pre-loaded blister-based reagent storage, for this research study, and fast optimisation cycles, reagents were introduced manually *via* pipetting to simulate blister actuation. Blister-based reagent release has been previously demonstrated on a related finger-actuated platform.^25^

### Extraction on a robotic platform

To evaluate and compare the performance and reproducibility of the device extraction method, automated robotic extraction was performed in addition to the standard bench extraction. An automated protocol was performed using the Opentrons Flex liquid handling platform (Opentrons, USA). This robotic benchmark enabled high-throughput comparison with both the manual benchtop and finger-actuated microfluidic methods. Automated workflows were developed using the Opentrons Protocol Designer for standardised deck layouts. The system utilised a single-channel P1000 and a P50 electronic pipettor. The Flex deck was configured with a magnetic block for bead separation and a heater-shaker module to automate the lysis, binding, wash and elution steps, directly mirroring the mechanical conditions of the manual benchtop protocol. To ensure high-quality recovery, the robot was programmed with specific “aspirate” and “dispense” speeds to prevent bead loss during the supernatant removal phases. A complete protocol script used for these extractions are available in SI 2.

### Quantitative PCR (qPCR) analysis on spiked water and human healthy human plasma samples

Recovery of cell-free DNA on the device or benchmark was quantified using qPCR with LINE-1 primers,^28^ a marker for genomic DNA abundance. Reactions were carried out in a final volume of 12.5 μL, comprising 200 nM forward and reverse primers, 1 μL of extracted eluate as template and Power SYBR® Green PCR Master Mix (Applied Biosystems). Thermal cycling was performed on a Bio-Rad CFX96 Real-Time PCR System under the following conditions: initial denaturation at 95 °C for 10 min, followed by 30 cycles of 95 °C for 15 s and 60 °C for 60 s. Melt-curve analysis was included to verify amplification specificity. Quantification cycle (Cq) values were obtained and compared with standard curves to calculate absolute cfDNA recovery from plasma samples processed using either by the benchtop protocol, robotic protocol or the microfluidic device.

### qPCR on clinical patient samples

In-house real-time PCR assays were performed for cytomegalovirus (CMV) and chikungunya virus (CHIKV) on nucleic acid extracted from clinical patient plasma samples. Real-time PCR for monkeypox virus (MPXV) was conducted on nucleic acid extracted from lesion swabs. The CMV and MPXV PCR assays utilised Precision Fast LR qPCR Master Mix (Primer Design, UK), with final concentrations of 0.9 μM for each forward and reverse primer and 0.2 μM for the probe in a 20 μL reaction volume. For the two-step CHIKV real-time PCR assay, cDNA was first synthesised using the SensiFAST cDNA Synthesis Kit (Meridian Bioscience, UK). The resulting cDNA was then added to a 20 μL PCR reaction containing CHIKV primers and probe at the same final concentrations described above. All PCR reactions and subsequent analyses were performed on an ABI 7500 FAST real-time PCR system under the following thermal cycling conditions: 95 °C for 2 minutes (1 cycle), followed by 45 cycles of 95 °C for 5 seconds and 60 °C for 30 seconds.^29^

#### Statistical analysis

Statistical analysis was performed using GraphPad Prism (version 10.0). Data are presented as mean ± standard deviation unless otherwise stated.

## Results and discussion

### Module validation

Prior to evaluation of the fully prototype (Fig. 3A), the functional modules were evaluated by separate testing. The first module was designed to test if the flow of fluids passed through two valves was operator-dependent (Fig. 3B.i and ii). A second module was designed to measure fluid recovery through a 90° valve (Fig. 3C.i and ii). This modular validation strategy enabled independent characterisation of operator variability and fluid displacement accuracy, respectively.

**Fig. 3:**
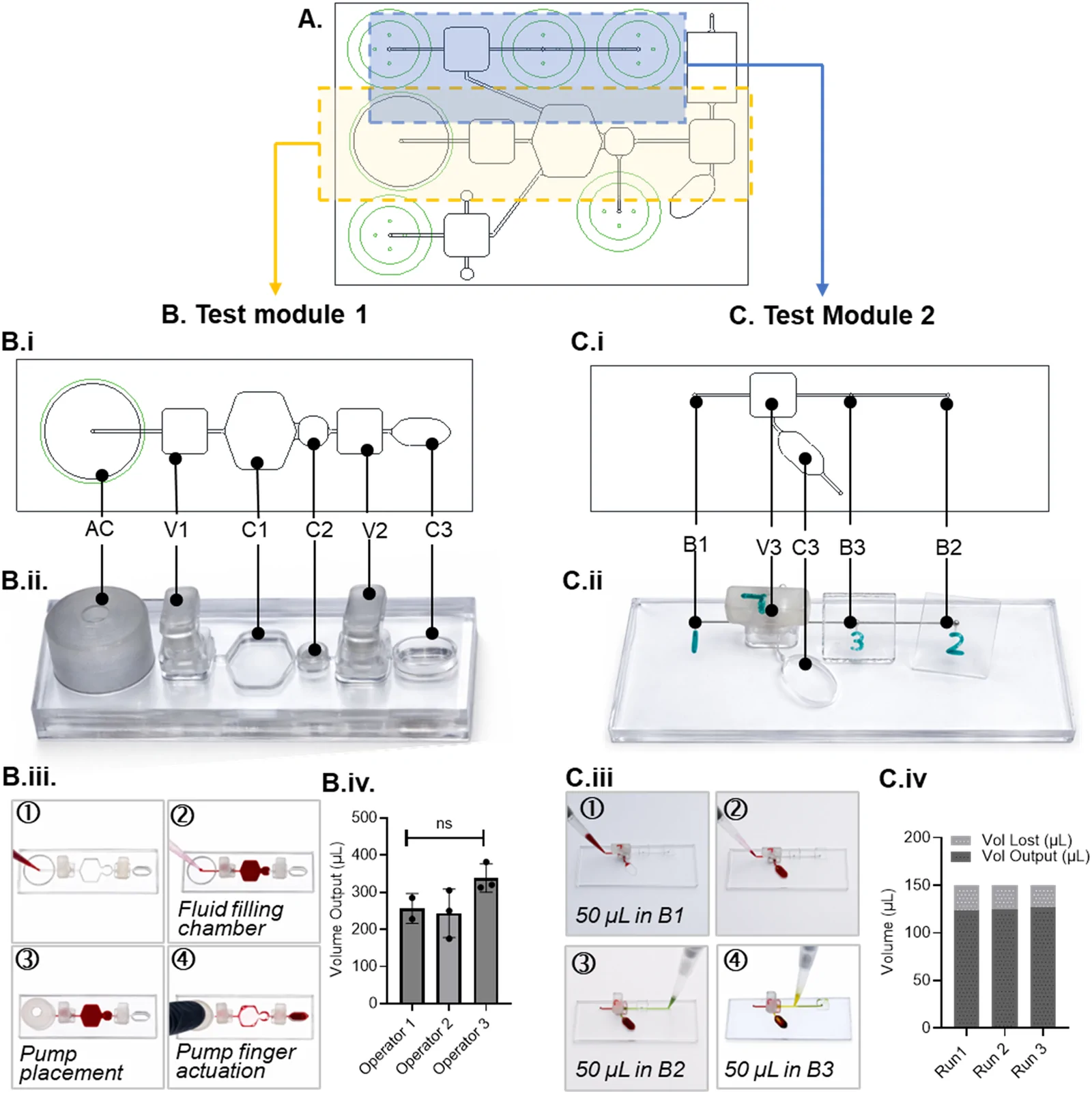
Validation of two different modules of the finger-actuated cfNA extraction device. A. Overall device layout and modular approach to modular design tests. B. Finger pump test module B.i CAD drawing and features. B.ii. Photograph of the fabricated finger-actuated pump module. B.iii: Operational sequence of the pump module. 450 μL of dyed solution was pipetted into the mixing chamber and pumped by three different operators. Fluid displacement into the elution chamber was measured as the volume of the dyed solution collected in the eluate chamber. B.iv. Quantification of pumped volumes per operator. No statistically significant differences were observed between operators (*p* > 0.05). C. Dead volume testing. C.i CAD schematic of isolated test module. C.ii Photograph of the test module. C.iii. Operational sequence of the test module. 50 μL of dyed solution was pipetted through the blister inlets into the collection chamber by valve actuation and the quantity of the dyed solution collected was then measured to calculate the fluid losses in the channels. C.iv. Quantification of total volume output and volume lost in the channels during independent experiments on individual chips (run).

Visual inspection during the operation of the pump test module confirmed complete chamber filling and consistent movement across repeated cycles (Fig. 3B.iii). Quantitative assessment of pumped volume demonstrated consistent transfer in replicate trials. Minor, non-significant, variations were observed between independent operators, likely attributable to differences in manual actuation timing and applied pressure (Fig. 3B.iv). It is possible that these variations, albeit minor and non-significant might affect the final cfNA recovery, and thus the final diagnostic sensitivity. However, the combination of the extraction device and a lateral flow device, or other passive detection device, is ultimately expected to be used in triage rather than highly quantitative workflows, therefore such variations should not prevent device use or significantly affect diagnostic performance. Nevertheless, further investigation will be needed.

The second module (Fig. 3C) was evaluated to determine the ability of the device to direct 50 μL of fluid from the three blister inlets (B1, B2, and B3) into a designated collection chamber (C3) using a multichannel 90° valve (V3) (Fig. 3C.iii). A set volume of 50 μL was pipetted through each blister inlet. The total volume collected from the collection chamber was then measured and compared to the total pipetted volume. On average 16.7% of the total volume was lost with minimal variation between runs (CV = 7.7%).

### Characterisation of the device using spiked water samples

Characterisation of the finger-actuated extraction device was conducted using 100 μL nuclease-free water spiked with defined human genomic DNA concentrations (TaqMan Control Genomic DNA, Thermofisher Scientific) (5–20 ng mL^−1^) to evaluate extraction performance. Eluted DNA concentrations were measured by qPCR. The raw Cq values are available in Fig. S1. For bench-top and device extraction, a clear and linear trend was observed, with increasing recoveries as input concentration increased from 5 to 20 ng mL^−1^ with *R*^2^ values of 0.86 for the device and 0.92 for the bench control extraction (Fig. 4A). This confirms correct assay behaviour and consistent recovery across the tested input range. The device demonstrated absolute recoveries of 84.6% on average across all spiking levels, against 90.7% on the bench (Fig. 4B). This also indicates effective nucleic acid capture, washing and elution on-chip. Finally, the Cq (difference in Cq values between device and bench) remained within ±0.5 cycles for three of the four concentrations tested (Fig. S1), demonstrating near-equivalent extraction performance: and the extracted amounts on bench and device demonstrated excellent agreement (*R*^2^ = 0.98) (Fig. 4C).

**Fig. 4:**
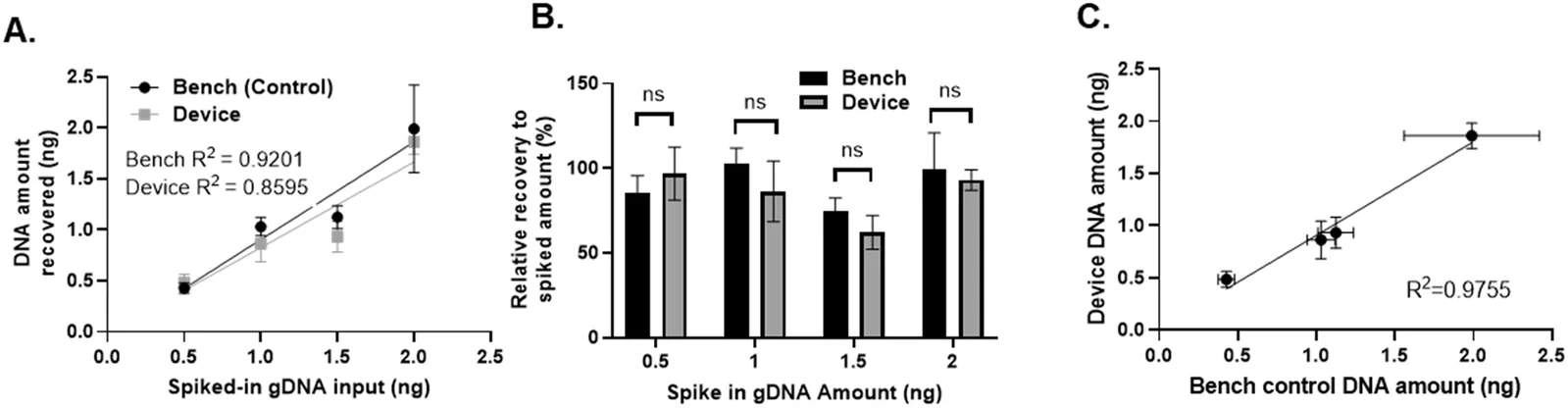
Characterisation of finger-actuated cfNA extraction device with known concentrations of analyte. Genomic DNA was spiked into water at four levels (5, 10, 15, and 20 ng mL^−1^) and processed using standard bench extraction (MagMAX™) and the microfluidic device to assess assay performance and linearity. A. Mean values of absolute recovery at each spike-in amount, demonstrating a concentration-dependent response across the tested range (*n* = 2). Bench and device assay linearity within the evaluated concentration range B. Relative recovery of bench and device extractions expressed as a percentage. C. Correlation between the recovered DNA amount on bench and device assays.

At the lowest spiked concentration (5 ng mL^−1^), the device recovered 13.5% more DNA than the bench. While this difference may partially reflect experimental variability, it indicates that the device does not compromise sensitivity at low nucleic acid concentrations or loss under lower input conditions on-chip. Overall, the microfluidic device demonstrated consistent behaviour, providing a baseline for subsequent testing in plasma matrices. While these recoveries are superior to the ones reported by Park *et al.*,^24^ they are not directly comparable, because they were determined using different DNA targets and spike units: HBV DNA copies per μL *versus* human genomic DNA ng mL^−1^.

### Magnetic beads volume optimisation

To determine the minimum magnetic bead volume required for efficient extraction, three bead input volumes (10, 50 and 100 μL) were evaluated extracting from 100 μL of spiked aqueous sample while all other reagent volumes and processing conditions were held constant. This optimisation was performed to reduce reagent consumption without compromising extraction performance. In principle, increasing magnetic bead volume is expected to enhance nucleic acid binding capacity and recovery. However, in practice, extraction efficiency increases with bead volume only up to a saturation point, beyond which additional beads can promote bead aggregation & clumping, inefficient washing and reduced elution efficiency, ultimately leading to lower recovered nucleic acid yield.

The results indicated that reducing the bead volume below a threshold reduced nucleic acid recovery, whereas increasing the bead volume beyond this threshold did not yield a proportional increase in recovery. Based on these findings, a bead volume of 50 μL was selected as the optimal condition, representing the lowest volume that maintained maximal recovery (Fig. S2).

### Device characterisation using healthy human plasma samples

Following validation in ideal aqueous samples, extraction performance across for three healthy human pooled plasma samples in three different sample volumes, was evaluated with conventional benchtop extraction, the finger-actuated microfluidic device extraction as well as an automated robotic extraction protocol. cfDNA concentrations were determined by qPCR following extractions and these results are summarised in Fig. 5. Raw Cq values, including failed extractions (not included in Fig. 5), are available in Fig. S3. Across all three pooled plasma samples (B011, B012, and B013), the finger-actuated microfluidic device demonstrated consistent nucleic acid recovery as compared to both manual benchtop and automated robotic extraction methods at input volumes of 100 μL and 75 μL. At a 100 μL input volume, recovery yields obtained with the device closely matched those of the bench methods, with only minor deviations observed across samples (non-significant statistical difference across all three pooled samples, Fig. 3A.i). As the input volume decreased to 75 μL and 50 μL, higher recovery was observed on the device compared to the bench and robotic extractions; however, this trend was particularly more pronounced at 50 μL, with statistical significance achieved for B013 on 75 μL (Fig. 3A.ii), and all samples on 50 μL (Fig. 3A.iii). The recovery of cfDNA across all three pooled samples from the device was 18, 62 and 552% superior to the bench for the 100, 75 and 50 μL starting samples, respectively. The robotic extraction was found to be inferior to both the bench and the device across all of the samples and volumes. It is generally well accepted that robotic extraction, although convenient and sometimes quicker, are not as good as manual extraction in terms of extraction yields. The viscosity of several extraction reagents, and the fact that magnetic beads settle quickly, make this particular assay more difficult to automate on fluid handling robots.

**Fig. 5:**
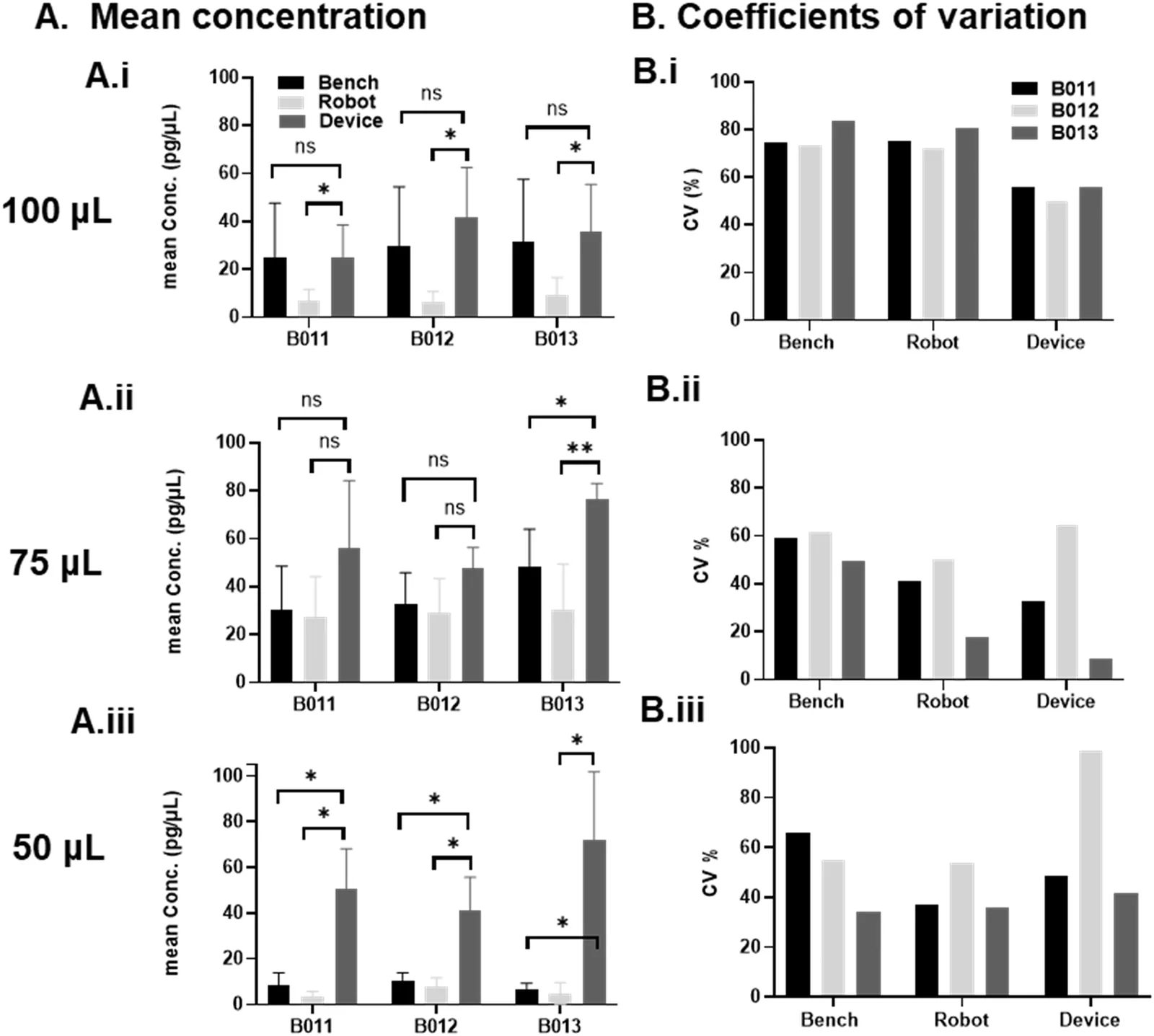
Characterisation of cfDNA extraction using human plasma samples in bench, finger-actuated device, and robotic extraction. A. Comparison of extraction performance across three different methods, manual benchtop, robotic and a finger-actuated device extraction, using plasma samples for three distinct pooled samples (B011, B012, B013). The evaluation was performed across three different starting sample volumes: A.i 100 μL, A.ii 75 μL and A.iii 50 μL. Bar charts showing the mean recovered concentration (pg μL^−1^) of the three targets (B011, B012, B013). Error bars indicate the standard deviation. The statistical test applied was unpaired Student-*t*-test with Welsh correction. Some outliers were removed from the bench and robot datasets but no outlier was removed from the device dataset. The raw Cq values of the entire dataset, including outliers are visible in Fig. S3. B. Coefficient of variation (%) for each extraction method for the three pooled samples in the three starting sample volumes: B.i 100 μL, B.ii 75 μL and B.iii 50 μL.

A critical metric for the clinical deployment of point-of-care microfluidic devices is the reproducibility of extraction, quantified by the coefficient of variation (CV). In our evaluation across the tested samples, the finger-actuated device exhibited variability that remained within established laboratory ranges for standard bead-based extractions, typically 20–80%.^30,31^ Interestingly, at the maximum tested input volume of 100 μL, the device achieved lower CVs values than both the manual benchtop and automated robotic reference methods. While the device exhibited slightly higher variability at lower input volumes (50 μL and 75 μL), this trend is expected; low-volume microfluidic extractions are inherently more sensitive to minute fluidic losses and manual handling variations during elution. When contextualising these results against recent literature, it is important to note that many platforms reporting highly consistent recoveries rely on heavily automated pumping mechanisms and process significantly larger sample volumes ranging from 500 μL up to 4 mL of plasma or whole blood.^32–34^ Operating at these macro-volumes naturally buffers against the microfluidic losses that impact CVs. It should be noted that multiple parameters such as incomplete reagent transfer, bead loss, and drying efficiency, whilst difficult to quantify independently, may directly affect the cfDNA extraction variability. Bead losses could be further mitigated by careful redesign of the reaction chamber with steps to prevent the beads escaping the chamber. Calculated dead volumes have been taken into account when adjusting the reagent volumes. Ultimately, a finger-actuated device might not be expected to perform at the same level of variability as a high-end lab equipment, a trade-off between simplicity, convenience and variability is expected. Overall, these results demonstrate that the finger-actuated platform achieves performance comparable to or better than established extraction methods in the 50 to 100 μL sample volume range.

To assess the agreement between the finger-actuated device and the reference extraction method, a Bland–Altman analysis was performed for each biological sample across all tested input volumes (50 μL, 75 μL, and 100 μL) (Fig. S4). In all cases, the data points fell within the 95% limits of agreement, indicating no systematic bias between the finger-actuated platform and the reference bench method. As expected from the previous analysis, the bias increased with the decreasing sample volumes. The absence of outliers beyond the limits of agreement further supports the reliability of the finger-actuated mechanism as a reproducible extraction approach.

Due to their intrinsic, manual features, finger-actuated devices are expected to suffer from operator variability. Here, several extractions were carried out by two operators on the same human plasma samples following the same set of instructions. Although a difference in average inter-operator concentration was measurable, this was found to be non-significantly different (Fig. S5). In the future the study could be further expanded to include more operators, with different level of familiarity with the device (*e.g.* experimented *vs.* novice operators) and understanding the impact of the variability of recovered cfDNA on different targets, detection platforms and clinical scenarios.

### Validation with clinical viral samples

To evaluate clinical applicability, the finger-actuated device was tested using clinical viral patient samples at the Victorian Infectious Diseases Reference Laboratory (VIDRL) at the Peter Doherty Institute, Melbourne, Australia.

This clinical pilot was designed as a proof-of-concept study to demonstrate feasibility with authentic patient samples and define the current operational limits of the device. The selected sample targets enabled evaluation of device performance across clinically relevant sample matrices (plasma and lesion samples) and both DNA and RNA viruses. The chosen patient samples had been tested positive for cytomegalovirus (CMV), monkeypox virus (MPXV) or chikungunya virus (CHIKV) using ISO15189 accredited assays. Absolute DNA quantification was not available in this clinical laboratory.

Initial experiments performed with a magnetic bead volume of 20 μL instead of the optimised 50 μL, yielded no detectable amplification for CHIKV and CMV samples. This suggests that residual ethanol carryover significantly inhibits downstream amplification in the microfluidic format for this particular application. However, following the use of 50 μL magnetic bead volume and the introduction of a quick heat-assisted drying step (approximately 2 min at 65 °C on heat block) successful amplification was achieved across all targets and extracted samples (Table 1). For CHIKV, the device (*n* = 2) yielded a mean Cq of 32.7 compared to 17.4 for the benchtop protocol, indicating reduced sensitivity from device eluates. CHIKV is an RNA virus. The device was not specifically developed for RNA extraction, which may explain the difference in performance compared with an RNA-optimised protocol. Whilst the extraction chemistry does not remove RNA, the non-specific RNA binding is expected to be greater compared to DNA.^35^ The RNA extraction could be further improved by optimising the reagents and reducing RNA binding through passivation. In contrast, CMV (*n* = 2) and MPXV (*n* = 1) showed close agreement between methods, with Cq differences of <2 cycles.

**Table 1:** Device characterisation with clinical viral samples. Comparison of cycle threshold (Cq) values for viral nucleic acid extraction performed using a standard benchtop method *versus* the tested device. The evaluation includes three clinical viral targets: chikungunya virus (RNA), cytomegalovirus (CMV, DNA), and monkeypox virus (DNA). No amplification were observed in negative controls up to 40 cycles

Viral target	Nucleic acid type	Sample type	Bench Cq	Device Cq	Observation
Chikungunya virus (CHIKV)	RNA	Plasma	17.4 (*n* = 2)	32.7 (*n* = 2)	Detectable
Cytomegalovirus (CMV)	DNA	Plasma	26.0 (*n* = 2)	27.8 (*n* = 2)	Comparable
Monkeypox virus (MPXV)	DNA	Lesion swab in buffer	26.6 (*n* = 2)	26.9 (*n* = 1)	Comparable

These results, whilst limited, demonstrate that the device can extract both DNA and RNA viral nucleic acids without extensive instrumentation. The higher Cq values observed for CHIKV and other targets can be attributed to reagent chemistry not specifically optimised for viral RNA recovery. Despite this, the finger-actuated platform successfully enabled the extraction and detection of both DNA and RNA viruses from plasma and lesion-derived samples without any additional sample pre-treatment, protocol modification, or extensive instrumentation, highlighting its versatility. Variability relative to laboratory workflows could be acceptable for qualitative or semi-quantitative point-of-care applications, particularly when weighed against the advantages of portability, operational simplicity and independence from laboratory infrastructure. It should be noted that, although not all isothermal amplification, PCR, or LFA assays require nucleic acid extraction, the most sensitive assays are expected to benefit from extraction to ensure functionality or improve sensitivity.^23,36^ More research is needed to establish which assays would best benefit such rapid field-ready extraction protocol. In addition, it should be noted that qPCR is used here as a characterisation tool only. We propose this device to be interfaced with simpler detection methods, such as LAMP or RPA assays to enable point-of-care use. This will be the subject of follow-on studies.

With regards to usability, we believe the device is intuitive and can be used by a fairly short list of instructions. A similar device, albeit with a smaller number of valves and blisters, was demonstrated by a ten year old child.^26^ The device usability is comparable to other field-based and finger-actuated DNA extraction solutions. Reboud *et al.*,^11^ proposed an extraction on an origami paper device. It has roughly similar number of steps, with similar complexity (the use of button and turning valves is replaced by having to fold the paper in precise steps, and then tear away part of the paper strip). Ultimately, the present device will not need the use of a pipette as in Reboud *et al.*, and will have all reagents on board. Another solution from Wang *et al.* included some similar ‘finger pumps’.^37^ The device use seems simpler at first (less number of steps), but the fabrication steps of their device are more complex, and the PDMS ‘buttons’ above the reagents are unlikely to provide the same vapor barrier for long-term storage than aluminium blisters. In addition, the device from Wang *et al.* include off-chip preparation steps. Furthermore, the number of valves in our device could be further reduced in future development.

## Conclusion

The finger-actuated microfluidic nucleic acid extraction device presented here offers a robust, relatively low-cost and instrument-free approach to upstream sample preparation for point-of-care molecular diagnostics.

Device performance was found to depend on key operational parameters, particularly magnetic bead volume and effective drying and ethanol removal, with a bead volume of 50 μL identified as optimal for maintaining high recovery while also minimising reagent use. The platform demonstrated nucleic acid recovery comparable to conventional benchtop methods, with Cq values from PCR quantification typically within ±0.5 cycles in controlled spike-in experiments. It also showed consistent recovery across plasma input volumes of 50–100 μL. In clinical viral samples, successful extraction and downstream amplification of both DNA and RNA targets were achieved, with Cq differences of <2 cycles for DNA viruses following optimisation.

By eliminating the need for external pumps, centrifugation and skilled laboratory handling, this platform represents a significant step towards fully integrated, portable sample-to-answer diagnostic systems. The ability to process clinically relevant samples using manual actuation alone demonstrates the feasibility of decentralised nucleic acid extraction without compromising analytical performance.

While this study focused only on plasma, future work could extend the platform to additional clinical sample types, including whole blood, respiratory, and urine specimens, and further optimise performance. Another possibility is expanding compatibility of the device and associated point-of-care detection with a broader range of nucleic acid targets for several other infectious or metabolic disorders (*e.g.* drug-induced liver injury, non-alcoholic fatty liver disease).

Overall, this work lays a foundation for scalable, user-friendly microfluidic extraction technologies and highlights the potential of finger-actuated systems to enable accessible molecular diagnostics in resource-limited settings and at the point of care. Further validation with larger clinical sample sets will be the focus of future studies.

## Ethical statement

Healthy human plasma samples for the device characterisation were sourced from the Scottish National Blood Transfusion Service (contract 18–20; Office for Research Ethics Committees Northern Ireland (ORECNI) ref. 18/NI/0148). Patient samples (plasma samples and lesion swabs) used in the clinical pilot study were obtained from the Victorian Infectious Diseases Reference Laboratory (VIDRL) at the Peter Doherty Institute and collected under ethical approval of Royal Melbourne Hospital Human Research Ethics Committee (RMH HREC 2020.179).

## Conflicts of interest

There are no conflict of interest to declare.

## Supplementary Material

LC-026-D6LC00439C-s001

LC-026-D6LC00439C-s002

## Data Availability

Data for this article, including raw PCR Cq values, and analysis file are available at FigShare at: https://doi.org/10.6084/m9.figshare.32298783 https://doi.org/10.6084/m9.figshare.32298810 Supplementary information is available. Supplementary Fig. S1–5 are provided in a PDF file. The code for the OpenTrons cfDNA extraction script is provided as a ZIP file. See DOI: https://doi.org/10.1039/d6lc00439c.
