# Supplementary material for: Finger-actuated microfluidic device for point-of-care circulating cell-free nucleic acid extraction
Source: Lab Chip. 2026 Aug 14;26(20):5401–11. doi: 10.1039/d6lc00439c (PMC13660751; doi:10.1039/d6lc00439c)
Supplement: Supplementary file 1 [file LC-026-D6LC00439C-s002.pdf]

Supplementary Information (SI) for Lab on a Chip.
This journal is © The Royal Society of Chemistry 2026

Supplementary Figures

Finger-Actuated Microfluidic Device for Point-of-Care Circulating Cell-free Nucleic Acid Extraction

Ghulam Musaddaq, Labibah Ehsan, Yano Yoga, Joshua Castillo, Linda Marriott, Nicholas R. Leslie, Deborah Williamson, Thomas Tran, Chuan Kok Lim, and Maïwenn Kersaudy-Kerhoas

Supplementary Information Figure S1

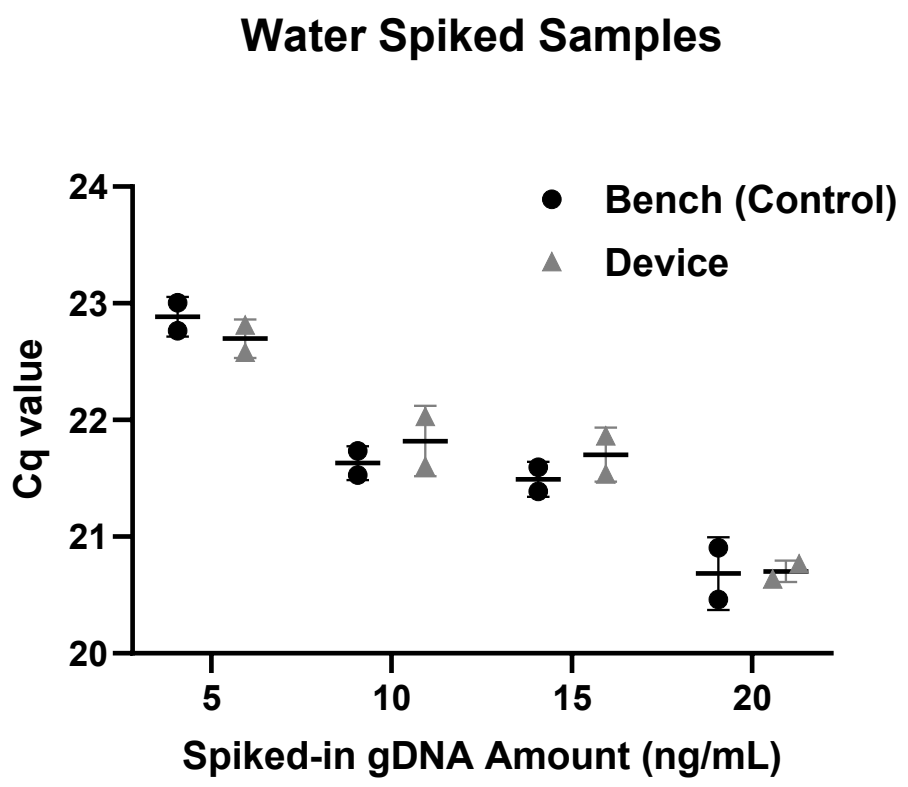

Figure S1*: Cq values and standard deviation for various spike-in concentration levels (ng/mL) N=2*

Supplementary Information Figure S2

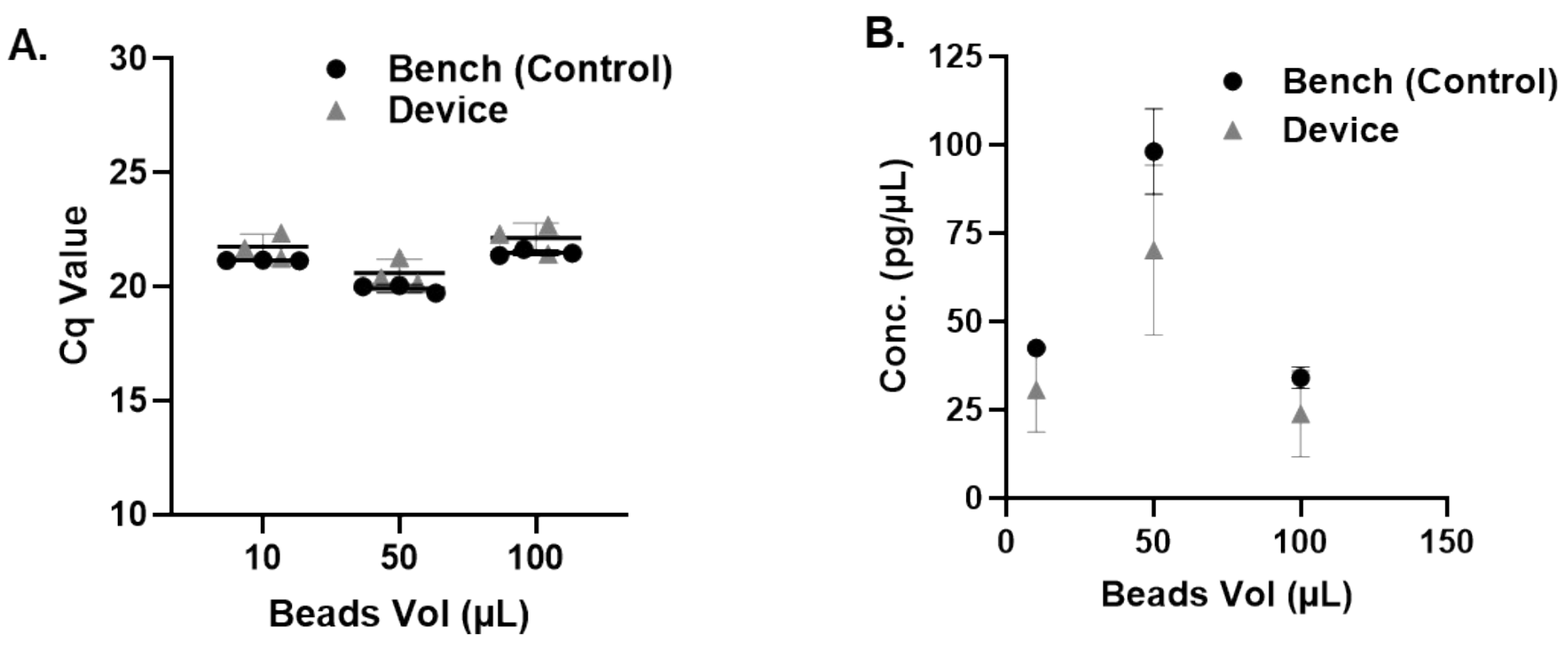

Figure S2. *Optimisation of magnetic bead volume for nucleic acid extraction. A. Raw Cq values. Consistent with this, the lowest Cq values were also observed at 50 µL, indicating improved nucleic acid recovery relative to 10 and 100 µL. B.Measured nucleic acid concentration (pg.µL* $^{-1}$*) obtained using the microfluidic device was compared with a benchtop control protocol across three magnetic bead input volumes (10, 50, and 100 µL). Maximal recovery was observed at a bead volume of 50 µL for both the bench and the device extraction.*

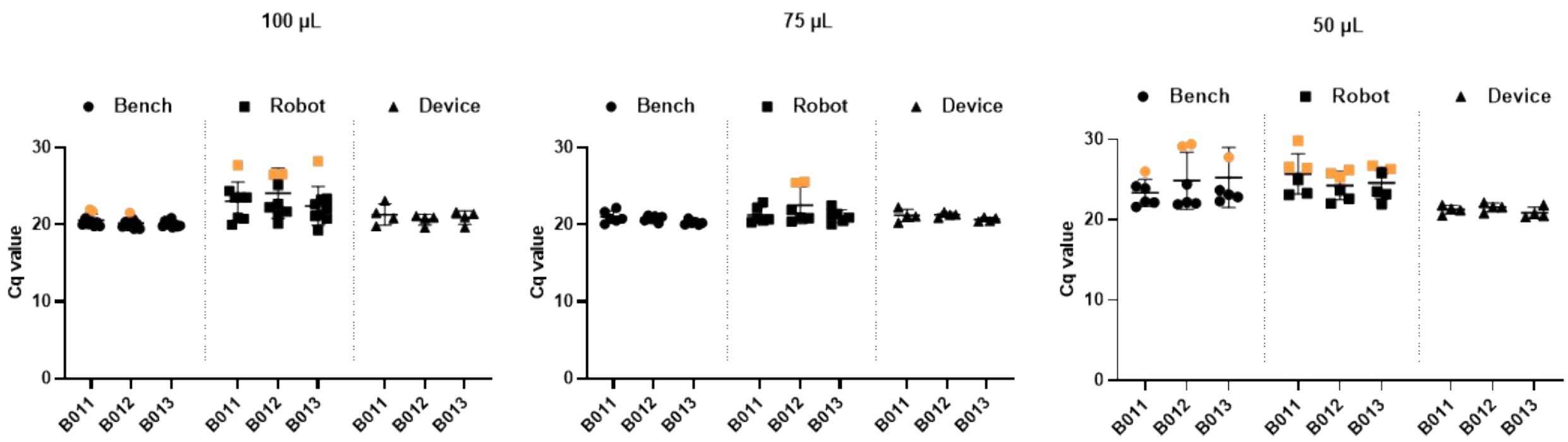

Supplementary Information Figure S3

Figure S3. *Raw individual Cq values for all extraction techniques, all pooled samples and all volumes. The outliers points excluded from analysis are marked in orange. The following rules are used to excludes points. High Ct values indicate low amounts of DNA in the sample. In this analysis, only samples with high Ct values (i.e. very low DNA recoveries) were excluded from Bench and Robot datasets. As a result, the revised datasets contained samples with comparatively higher DNA recoveries overall. The exclusion criteria for the 50 and 75 µl samples on Bench and Robot: concentrations<1pg/ul. The exclusion criteria for the 100 ul samples on bench: conc.<5pg/ul and on Robot: conc.<1pg/ul. Note no sample were excluded from the Device dataset.*

Supplementary Information Figure S4

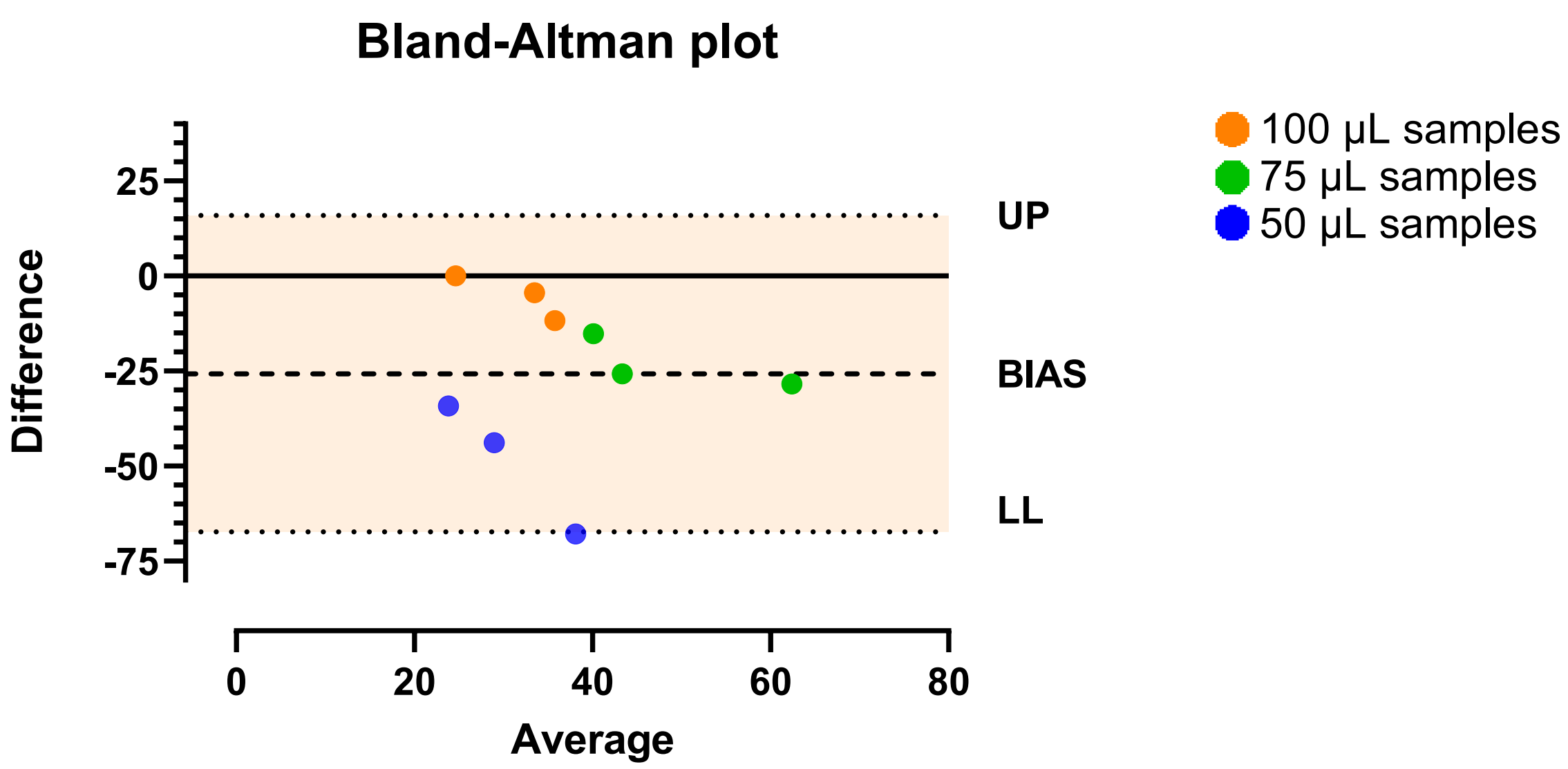

Figure S4: Bland Altman plots of microfluidic device extractions and control bench extractions across three pooled samples and three sample volumes. UP = Upper Limit of confidence LL= Lower Limit of confidence.

Supplementary Information Figure S5

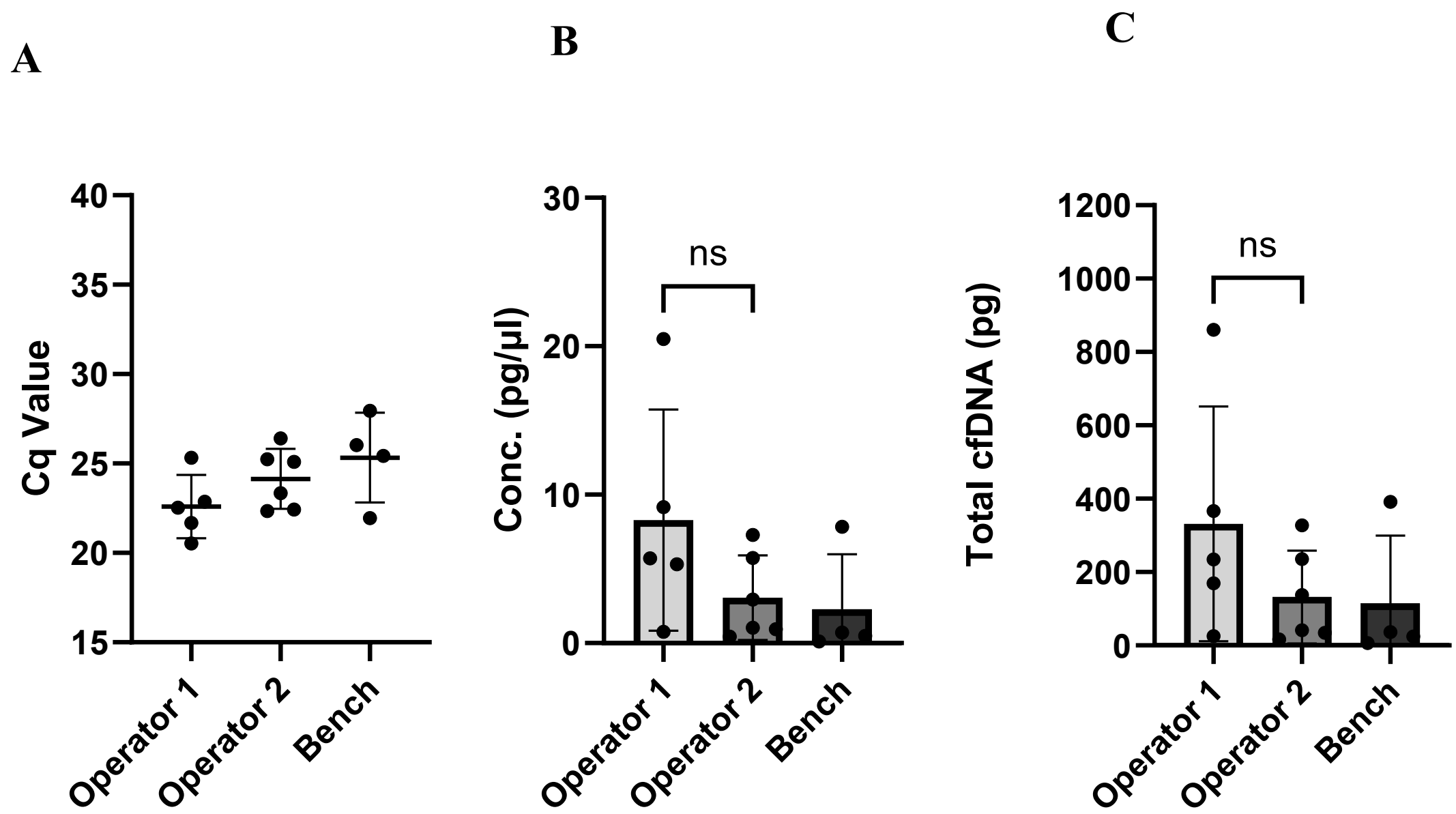

Figure S5. Comparison of cfDNA recovery by different operators on plasma sample from a single donor (B004). (A) Cq values and (B) cfDNA concentration (pg/µL) following extraction by two independent operators and the bench control. (c) Total cfDNA amount recovered. This was obtained by multiplying the concentration by the recovered eluate volume. Data are presented as mean ± SD (Operator 1, n = 5; Operator 2, n = 6; Bench control, n = 4). No statistically significant difference was observed between Operator 1 and Operator 2 ($P > 0.05$). Increased variability between the extraction replicates was observed because some of the device valve housings were fabricated using a different resin (Flexible 50A Resin, Form 3), resulting in unexpected leakage issues during device operation by the operators. The other valve housings in these experiments were made either with the stated Method or with PDMS following the method stated in Haque et al, 2022.
